# Identifying the Association of Contrast Enhancement with Vascular Endothelia Growth Factor Expression in Anaplastic Gliomas: A Volumetric Magnetic Resonance Imaging Analysis

**DOI:** 10.1371/journal.pone.0121380

**Published:** 2015-03-30

**Authors:** Yinyan Wang, Kai Wang, Hongming Li, Jiangfei Wang, Lei Wang, Jianping Dai, Tao Jiang, Jun Ma

**Affiliations:** 1 Beijing Neurosurgical Institute, Capital Medical University, Beijing, China; 2 Department of Neurosurgery, Beijing Tian Tan Hospital, Capital Medical University, Beijing, China; 3 Department of Neuroradiology, Beijing Tian Tan Hospital, Capital Medical University, Beijing, China; 4 Institute of Automation, Chinese Academy of Sciences, Beijing, China; 5 China National Clinical Research Center for Neurological Diseases, Beijing, China; 6 Center of Brain Tumor, Beijing Institute for Brain Disorders, Beijing, China; The University of Hong Kong, CHINA

## Abstract

Contrast enhancement is a crucial radiologic feature of malignant brain tumors, which are associated with genetic changes of the tumor. The purpose of the current study was to investigate the potential relationship among tumor contrast enhancement with MR imaging, vascular endothelial growth factor (VEGF) expression, and survival outcome in anaplastic gliomas. MR images from 240 patients with histologically confirmed anaplastic gliomas were retrospectively analyzed. The volumes of T2 hyperintense, contrast enhanced regions and necrotic regions on postcontrast T1-weighted images were measured. The ratio of the enhanced volume to necrotic volume was compared between patients with high versus low levels of VEGF expression and was further used in the survival analysis. The volumetric ratio of enhancement to necrosis was significantly higher in patients with low VEGF expression than in those with high VEGF expression (Mann-Whitney, *p* = 0.009). In addition, the enhancement/necrosis ratio was identified as a significant predictor of progression-free survival (Cox regression model, *p* = 0.004) and overall survival (Cox regression model, *p* = 0.006) in the multivariate analysis. These results suggest that the volumetric ratio of enhancement to necrosis could serve as a noninvasive radiographic marker associated with VEGF expression and that this ratio is an independent predictor for progression-free survival and overall survival in patients with anaplastic gliomas.

## Introduction

Anaplastic gliomas (AG) are classified by the World Health Organization (WHO) as malignant tumors (grade III) and include anaplastic astrocytoma (AA), anaplastic oligodendroglioma (AO), and anaplastic oligoastrocytoma (AOA). These highly aggressive tumors often occur in adults between 40 to 50 years of age and typically recur or progress to grade IV glioblastomas within several years of diagnosis [1]. Despite surgical treatment and adjuvant therapy, the 5-year overall survival (OS) rate for patients with AG is estimated to be less than 40% [2]. Established prognostic factors include the patient’s age, tumor grade and histologic features, extent of surgical resection, and KPS. The role of radiologic features in determining the prognosis of patients with AG has been rarely investigated.

Contrast enhancement is one of the major MR characteristics of high-grade gliomas, but the degree of contrast enhancement is variable among individuals. When a tumor presents with enhancement, this presentation generally indicates that the tumor grew rapidly and that the blood-brain barrier was disrupted, which make gross-total resection difficult and leads to an unsatisfactory prognosis [3–5]. Previous studies suggested that the radiologic manifestations of the human glioma may be associated with the expression of tumor-specific molecular markers [6–8]. Nevertheless, whether the contrast enhancement of a tumor on an MR image correlates to genetic changes in the tumor is still unclear in patients with AG. Vascular endothelial growth factor (VEGF) is a well-known tumor-related biomarker that regulates pathological angiogenesis [9]. In vivo, VEGF expression promotes angiogenesis and the permeabilization of blood vessels, which is associated with the degree of enhancement in gliomas [10]. VEGF can also serve as an independent indicator for the prognosis of patients with liver cancer [11], breast cancer [12,13] and gastric cancer [14,15]. However, the relationship between VEGF and the radiologic enhancement of AG in humans has not been investigated.

The present study investigated the underlying relationships among radiologic contrast enhancement, VEGF expression, and survival outcome for patients with AG by performing a volumetric analysis of structural MR images. Our results show that tumor enhancement was associated with the VEGF expression level and was an independent prognostic factor for patients with AG. These findings increase our understanding of the association between molecular changes with radiological features of AG and imply a role for preoperative MR imaging in survival prediction.

## Materials and Methods

### Patients

A total of 240 patients who underwent primary surgical resection at our institute between October 2007 and April 2010 were retrospectively reviewed. All patients with histologically confirmed AG (WHO Grade III) were included in this study. The inclusion criteria were as follows: 1) age ≥ 18 years old; 2) presurgical MR imaging available, including T1-weighted, T2-weighted and postcontrast T1-weighted; 3) no previous diagnosis of any brain tumor; and 4) available tumor sample for immunohistochemical examination of VEGF expression. Patients were excluded if they had undergone any prior craniotomy or biopsy of the tumor. The histopathologic diagnosis was evaluated and confirmed by two independent neuropathologists who were blind to the patients’ information. The clinical information and radiographic features were derived from hospital documents.

### Treatment

The extent of surgical resection was assessed by postoperative MR images. Gross total resection (GTR) was defined as no visible contrast-enhancing tumor on postoperative MR images within 72 hours after surgery, or removal of all abnormal hyperintense changes on preoperative MR images for tumors not demonstrating contrast enhancement [16]. Resections that were not GTR were defined as residual tumor (<GTR). During the study period, 197 cases received adjuvant therapy after tumor resection, including radiotherapy and chemotherapy.

### Magnetic resonance imaging

Presurgical, postcontrast, T1-weighted images and T2-weighted images were acquired using a standard pulse sequence on a Magnetom Trio 3T (Siemens AG, Erlangen, Germany) scanner. Postcontrast, T1-weighted images were acquired after the injection of gadopentetate dimeglumine (Ga-DTPA Injection, Beilu Pharma), which was administered at 0.1 mmol/kg, using an echo time (TE) of 15 ms, a repetition time (TR) of 450 ms, and a slice thickness of 5 mm. The T2-weighted images were acquired using a TE of 140 ms, TR of 8000 ms, and slice thickness of 5 mm. The contrast agent used was acquired from the same pharmaceutical company (gadopentetate dimeglumine via injection; Ga-DTPA Injection, Beilu Pharma). In addition, the time interval between the injection of contrast agent and the beginning of the acquisition of the axial, contrast-enhanced, T1-weighted images was limited to 75–85 seconds.

### Regions of interest

**Fig 1:**
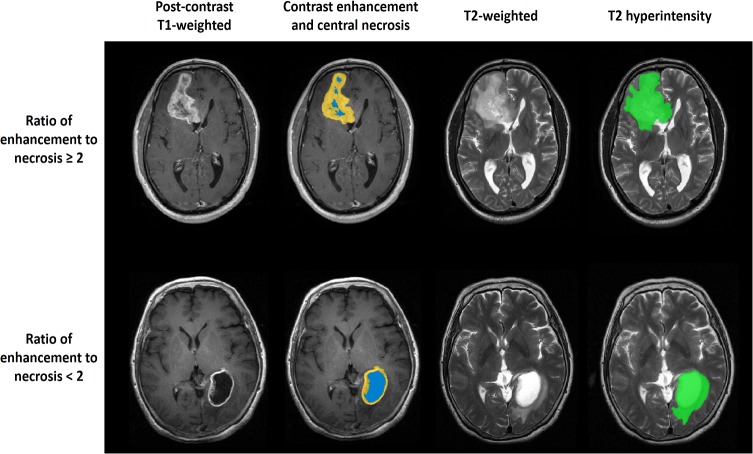
Anatomic MR images for anaplastic gliomas with different ratios of enhancement to necrosis (≥ 2 and < 2). The areas of contrast enhancement are marked in yellow; areas of central necrosis are marked in blue; areas of T2 hyperintensity (exclusion of contrast enhancement) are marked in green.

The regions of interest were manually delineated by two experienced neuroradiologists (Q. Chen and X. Chen, 14 and 12 years of experience in diagnosis using brain MRI, respectively) using MRIcro software (http://www.mccauslandcenter.sc.edu/mricro) and included 1) T2 hyperintensity on the T2-weighted images, 2) contrast enhancement on the postcontrast T1-weighted images, and 3) central necrosis on the postcontrast T1-weighted images (Fig. 1). The area of tumor contrast enhancement did not include the necrosis in this study. A third neuroradiologist (J. Ma, 25 years of experience) re-examined the images and determined the image for use when a > 5% inconsistency was found between the masks from the two previous neuroradiologists. All investigators were blinded to the patients’ clinical information. In addition, the volumetric ratio of enhancement to T2 hyperintensity, necrosis to T2 hyperintensity, and enhancement to necrosis were computed and used as radiologic markers for patients with AG.

### Immunohistochemistry

Immunohistochemical examination for VEGF was performed in all patients. Samples with more than 80% tumor cell consistency were selected for further assessment based on the morphology of the cells. Briefly, the tumor tissues were formalin-fixed and embedded in paraffin. For methylation detection, 5 μm sections were incubated with monoclonal antibodies against VEGF (Santa Cruz Biotechnology, Santa Cruz, CA). Control samples without primary antibody and positive control tissues were included in all experiments to ensure staining quality. VEGF was scored using a four level grading criterion: (-) indicated no or rare expression (< 5% positive cells); (+) indicated mild expression (6–25% positive cells); (++) indicated moderate expression (26–50% positive cells); and (+++) indicated strong expression (> 50% positive cells). Each slide stained for VEGF was individually reviewed and scored by two independent observers. AGs were classified into two groups based on the level of VEGF expression. The low expression group (LEG) was defined as VEGF (- and +), and the high expression group (HEG) was defined as VEGF (++ and +++) (S1 Fig.). Discrepancies in scoring between the two observers were resolved by additional review of the specimens and discussion between the reviewers until a consensus was achieved.

### Ethics statement

The study was approved by the institutional review board of Beijing Tian Tan Hospital and written consent form was obtained from all participants. The individual in this manuscript has given written informed consent to publish these case details.

### Statistical analysis

Because neither the volume nor the volumetric ratio data followed a normal distribution (Kolmogorov-Smirnov, *p* < 0.01 for all categories), nonparametric statistical tests were used. Specifically, a Mann-Whitney test was used to examine the difference in volumes and volumetric ratios among the different pathologic subtypes and VEGF expression grades. Additionally, a log-rank analysis on the Kaplan-Meier data was performed to compare progression-free survival (PFS), which refers to the time interval from primary surgery to tumor re-occurrence, and OS among the different groups. Factors with significance were further analyzed with a multivariate survival analysis based on the Cox proportional hazards ratio model.

## Results

### Patient characteristics

The overall information for the 240 patients is summarized in Table 1. Contrast enhancement on MR images was observed in 171 patients. Among them, 116 (67.8%) showed a single enhanced focus. For 157 patients with necrotic regions on the postcontrast T1-weighted images, 114 (72.6%) had a volumetric ratio of enhancement to necrosis higher than 2.0. A total of 161 patients received a GTR; 78 patients did not undergo GTR and had residual tumor. 197 patients received adjuvant therapy after surgical resection, including radiotherapy or chemotherapy, while the remaining 43 patients did not receive any adjuvant treatment. The interval between the preoperative MR examination and the pathological diagnosis ranged from 7 to 39 days, with a median time of 22 days.

**Table 1:** Characteristics of overall patients with anaplastic gliomas.

**Characteristics**	**Number (%)**
**Number of patients**	240
**Age**	
Median (Range)	43 (18–87 yr)
**Gender**	
Male	139 (57.9)
Female	101 (42.1)
**KPS**	
≥ 80	189 (78.6)
< 80	51 (21.4)
**Enhancement**	
Yes	171 (71.3)
No	69 (28.7)
**Enhancing foci**	*n = 171*
Single focus	116 (67.8)
Multi foci (≥ 2)	55 (32.2)
**Enhancement/necrosis**	*n = 157*
≥ 2	114 (72.6)
< 2	43 (27.4)
**Extent of resection**	
GTR	161 (67.1)
<GTR	79 (32.9)
**Adjuvant therapy**	
**Yes**	197 (82.1)
**No**	43 (17.9)
**VEGF expression**	
LEG^a^	132 (55.0)
HEG^b^	108 (45.0)
**Pathology**	
Anaplastic astrocytoma	67 (27.9)
Anaplastic oligodendroglioma	40 (16.6)
Anaplastic oligodendroastrocytoma	133 (55.5)

^a^LEG = VEGF (-) and VEGF (+)

^b^HEG = VEGF (++) and VEGF (+++)

Abbreviations: LEG, low expression group; HEG, high expression group; KPS, Karnofsky performance status score; VEGF, vascular endothelia growth factor.

### MR volumetric analysis with VEGF expression

**Fig 2:**
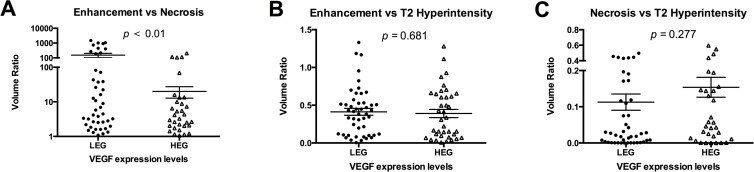
Volumetric ratios of enhancement to necrosis in anaplastic gliomas with different VEGF expression levels. (A) Ratio of enhancement volume to necrosis volume in the low VEGF expression group (LEG) and high VEGF expression group (HEG) (Mann-Whitney, *p* = 0.009). (B) Ratio of enhancement volume to T2 hyperintensity volume in the low VEGF expression group and high VEGF expression group (Mann-Whitney, *p* = 0.681). (C) The volumetric ratios of necrosis to T2 hyperintensity in the low VEGF expression group and high VEGF expression group were not significantly different (Mann-Whitney, *p* = 0.277).

The mean volume of the T2 hyperintensity was 119.7 cm^3^ for the LEG and 107.9 cm^3^ for HEG (*p* = 0.354). Neither the volume of contrast enhancement nor the volume of necrosis was different between the LEG and HEG (*p* = 0.650 and *p* = 0.258, respectively) (S2 Fig.). When combining these volumetric features by calculating the volumetric ratio of contrast enhancement to T2 hyperintensity, necrosis to T2 hyperintensity and enhancement to necrosis, a significantly higher ratio of enhancement to necrosis was identified in the LEG compared with the HEG (*p =* 0.009). The volumetric ratios of contrast enhancement to T2 hyperintensity (*p* = 0.681) and necrosis to T2 hyperintensity (*p* = 0.277) were not significantly different between the LEG and HEG (Fig. 2). Similarly, no significant differences were identified in the enhancement/necrosis ratios among the three pathologic tumor subtypes (S3 Fig.). Among the 171 tumors with significant enhancement, 14 tumors showed no apparent necrotic regions on the MR images. In this case, the enhancement/necrosis ratio of these tumors were considered as infinity and also included in the quantitative volumetric analysis.

### Prognostic factors for survival

**Fig 3:**
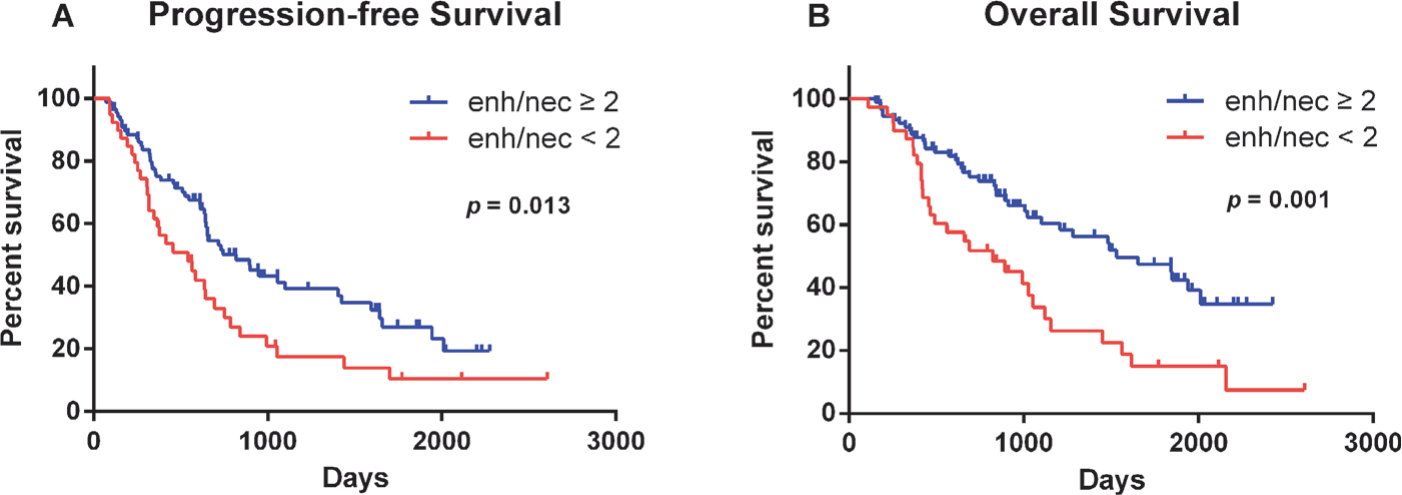
Kaplan-Meier estimates of survivals using volumetric ratio of enhancement to necrosis. Log-rank analysis indicated (A) a significant PFS advantage (log-rank, p = 0.013) and (B) a significant OS advantage (log-rank, p = 0.001) for patients with anaplastic gliomas with a high volumetric ratio (≥ 2) of enhancement to necrosis.

At the time of analysis, 56 of 240 patients (23.3%) remained alive, with a median follow-up of 49 months (range, 37–88 months). Thirty-five patients were lost to follow-up. In the univariate analysis, the age at diagnosis (*p* = 0.019), preoperative KPS (*p* = 0.001), extent of resection (*p* = 0.009), volumetric ratio of enhancement to necrosis (*p* = 0.013), oligodendroglial component (*p* = 0.025), and radiotherapy (*p* = 0.017) were identified as prognostic factors for PFS (Table 2). Other characteristics, including gender, contrast enhancement, multi-enhancing foci, VEGF expression, and chemotherapy did not have prognostic value. For the OS, the age at diagnosis (*p* = 0.027), preoperative KPS (*p* = 0.001), resection extent (*p* = 0.002), ratio of enhancement to necrosis (*p* = 0.001), and radiotherapy (*p* = 0.029) were identified as significant prognostic factors in the univariate analysis (Table 2). In the multivariate Cox regression analysis, age ≥ 50 (*p* = 0.010, HR = 1.897, 95% CI: 1.168–3.079), preoperative KPS < 80 (*p* = 0.001, HR = 3.173, 95% CI: 1.324–5.561), enhancement/necrosis ratio < 2 (*p* = 0.004, HR = 2.015, 95% CI: 1.247–3.258), and non-GTR (*p* = 0.012, HR = 1.780, 95% CI: 1.136–2.788) were indicators of poor prognosis for PFS. These four factors were also negative prognostic indicators for OS. Patients with age ≥ 50 (*p* = 0.019, HR = 1.854, 95% CI: 1.106–3.106), preoperative KPS < 80 (*p* = 0.008, HR = 2.359, 95% CI: 1.274–5.498), enhancement/necrosis ratio < 2 (*p* = 0.006, HR = 2.080, 95% CI: 1.240–3.491), and non-GTR (*p* = 0.019, HR = 1.831, 95% CI: 1.105–3.034) had a shorter OS (Table 3). Besides, radiotherapy was a protective factor for PFS (*p* = 0.022, HR = 0.692, 95% CI: 0.353–0.927) and OS (*p* = 0.032, HR = 0.715, 95% CI: 0.481–0.972) in multivariate analysis. Specifically, the Kaplan-Meier curve shows that patients with a higher (≥ 2) volumetric ratio of enhancement to necrosis were more likely to have a longer PFS and OS than those with a lower (< 2) volumetric ratio (Fig. 3).

**Table 2:** Univariate analysis of survival outcomes.

**Predictors**	**PFS**			**OS**		
	***p* value**	**HR**	**95% CI**	***p* value**	**HR**	**95% CI**
Age ≥ 50	0.019	1.639	1.080–2.487	0.027	1.691	1.062–2.693
Gender (M)	0.378	0.840	0.571–1.237	0.989	0.997	0.639–1.555
KPS < 80	0.001	2.748	1.253–3.570	0.001	2.916	1.519–3.326
< GTR	0.009	1.658	1.133–2.428	0.002	2.008	1.304–3.092
Enhancement	0.119	1.857	0.853–4.046	0.119	1.857	0.853–4.046
Enhancement/necrosis < 2	0.013	1.879	1.205–2.931	0.001	2.289	1.398–3.746
Multi-enhancing foci	0.212	1.336	0.848–2.104	0.154	1.450	0.870–2.415
VEGF (LEG/HEG)	0.610	1.104	0.688–1.890	0.621	1.156	0.651–2.053
Oligodendroglial component	0.025	0.563	0.340–0.931	0.064	0.579	0.325–1.033
Radiotherapy	0.017	0.718	0.354–0.921	0.029	0.649	0.508–0.982
Chemotherapy	0.136	0.723	0.542–1.057	0.140	0.748	0.529–1.096.

Abbreviations: HR, hazard ratio; CI, confidence interval; GTR = Gross total resection; PFS, progression free survival; OS, overall survival.

**Table 3:** Multivariate analysis of survival outcomes.

**Predictor**	***p* value** ^†^	**HR**	**95% CI**
**PFS**			
Age ≥ 50	0.010	1.897	1.168–3.079
KPS < 80	0.001	3.173	1.324–5.561
Enhancement/necrosis< 2	0.004	2.015	1.247–3.258
< GTR	0.012	1.780	1.136–2.788
Radiotherapy	0.022	0.692	0.353–0.927
**OS**			
Age ≥ 50	0.019	1.854	1.106–3.106
KPS < 80	0.008	2.359	1.274–5.498
Enhancement/necrosis< 2	0.006	2.080	1.240–3.491
< GTR	0.019	1.831	1.105–3.034
Radiotherapy	0.032	0.715	0.481–0.972

^†^Cox proportional hazard regression analyses.

A *p* value of 0.05 denoted significance.

Abbreviations: HR, hazard ratio; CI, confidence interval; GTR = Gross total resection; PFS, progression free survival; OS, overall survival.

## Discussion

With performing a volumetric neuroimaging analysis in a large patient cohort, the current study investigated the association between radiologic features, tumor-specific molecular markers (VEGF), and survival outcomes in patients with AG. Our results suggest that tumor enhancement was associated with the VEGF expression level and the ratio of enhancement to necrosis was an independent prognostic factor for patients with AG.

Several studies have attempted to correlate the MR enhancement of tumors with tumor-related biomarkers and survival outcomes. It was revealed that *IDH1* mutated glioma was more likely to present a less (or none) contrast enhancement, and a larger tumor size [17,18]. Besides, a previous study demonstrated that the tumor margin and intratumoral signal have prognostic values as surrogate markers for molecular characteristics in AOs [19]. In addition, the CT enhancement pattern and tumor proliferation index were identified as independent predictors of survival in a homogeneous series of patients with anaplastic gliomas [4,20]. One study suggested that occult postcontrast enhancement may be an MRI marker for angiogenesis in pediatric pontine glioma [21]. There were other studies found that dynamic contrast enhancement MRI parameters were related to VEGF and microvessel density (MVD) and that the mean and peak intensity of contrast enhanced ultrasound were significantly associated with MVD counts and VEGF expression in breast cancer; besides, VEGF may also enhance the hepatic mass with a contrast agent during CT [22–24]. Furthermore, it showed that the VEGF expression was correlated with the ^18^FDG uptake on PET-CT and with the blood volume on CT perfusion imaging in non-small cell lung cancer [25,26]. However, the relationship between MR contrast enhancement and VEGF expression has been rarely investigated in patients with AG.

In the present study, no significant difference was identified in the volume of contrast enhancement between the LEG and HEG. When comparing the ratio of enhancement to necrosis between these two groups, it was found that the HEG had a significantly lower volumetric ratio of enhancement to necrosis (*p =* 0.009), indicating that tumor with higher level of VEGF expression was prone to produce necrosis area. A previous study suggested that tumor cells are frequently deprived of oxygen due to the rapid cell proliferation accompanying tumor growth. This process leads to cell necrosis, which often occurs in the central region of the tumor area. Meanwhile, high levels of VEGF expression are found in the hypoxic regions that are usually located near the necrotic areas within the tumors [27]. This evidence supports our finding that the HEG was associated with a lower ratio of enhancement to necrosis than the LEG.

Recent studies showed that dynamic contrast-enhanced MR images could be associated with molecular markers of hypoxia and proliferation in specific areas of gliomas and rectal cancer [28,29]. In animal experiments, contrast enhancement represents vascular permeability and is only observed in highly angiogenic tumors with high VEGF expression [30]. VEGF induces permeabilization and the formation of fenestrations in blood vessels [31,32]. When an endothelial cell is damaged following hypoxic conditions, an MR contrast agent can extravagate, leading to the accumulation of contrast agent in the tumor tissue that causes abnormal enhancement. Thus, neovascular permeability is associated with the enhancement degree of gliomas [10].

As in previous studies of AG patients, age, preoperative KPS score, ring-like enhancement on CT, proliferation index, duration of symptoms, pre-irradiation performance status, tumor histology, and extent of resection were all independent variables that influenced survival [4,33]. The results of the current study suggests that age, KPS score, volumetric ratio of enhancement to necrosis, extent of resection, and radiotherapy are prognostic factors for both PFS and OS in patients with AG. As previously reported, the presence of an oligodendroglial component may also contribute to a favorable prognosis [34–36]. In this study, the oligodendroglial component was a significant factor for PFS (*p =* 0.025) but was not for OS (*p =* 0.064) in the univariate analysis. Meanwhile, it also was not prognostic factor in the multivariate analysis. In addition, the current study found that the ratio of enhancement volume to necrosis volume on preoperative structural MR images was a significant predictor of survival that may be used as a preoperative biomarker in clinical practice. Rapid proliferation and the resulting necrosis represent malignant behavior, which may lead to an unsatisfactory prognostic survival. Therefore, faster growing tumors are more likely to present with lower ratios of enhancement to necrosis, which may consequently influence the prognosis for patients with AG. Previous studies suggested that high VEGF mRNA expression was associated with poor clinical outcomes [37], while the genetic variation in VEGF and its receptor predicted a longer survival in patients with glioblastoma [38]. Meanwhile, the VEGF expression level could serve as an independent prognostic factor for patients with liver or breast cancer [11,13,15]. Although VEGF expression failed to present as a prognostic factor for the outcome of patients with AG, our results suggested that the level of VEGF expression was associated with enhancement/necrosis ratio, which was an independent factor of prognosis. Based on these findings, it was plausible to hypothesize that the VEGF expression may have something to do with the outcome of patients harboring AG, which needs further investigation.

Several limitations should be considered for this study. Distinguishing tumors from the area of edema was challenging. T2 hyperintensity was used in this study to represent the abnormal areas detectable on the MR image, which may include areas of edema and gliosis with sparse tumor cells in addition to the solid tumor area. Despite these limitations, this study provides quantitative evidence that suggests a relationship among contrast enhancement, molecular VEGF expression and survival outcome, and supports a role for radiographic features in predicting the survival of AG patients. In addition to AG, the prognostic value of the MR contrast enhancement for other types of brain tumors would be of interest for future investigations.

## Conclusions

In summary, this study retrospectively analyzed 240 patients with AG. A volumetric analysis was performed for all post-contrast, T1-weighted images and T2-weighted images. The volumetric ratio of enhancement to necrosis was associated with VEGF expression. In addition, an age ≤ 50 at the time of tumor diagnosis, a preoperative KPS score, extent of resection, a high volumetric ratio (> 2) of enhancement to necrosis, and radiotherapy were independent predictors of survival in patients with AG. Furthermore, there is a great clinical need for the classification of AG for prognosis based on their MRI features.

## Supporting Information

S1 Fig Semiquantitative analysis of VEGF expression by immunohistochemical staining. (A) Negative (-); (B) weakly positive (+); (C) positive (++); (D) strongly positive (+++). Magnification: ×200.
(TIF)

S2 Fig Volumetric analysis of T2 hyperintensity, enhancement and necrosis regions in anaplastic gliomas with different VEGF expression levels. (A) The volumes of T2 hyperintensity for the low VEGF expression group (LEG) and high VEGF expression group (HEG) were not significantly different (Mann-Whitney, *p* = 0.354). (B) The volumes of enhancement for the low VEGF expression group and high VEGF expression group were not different (Mann-Whitney, *p* = 0.650). (C) The volumes of necrosis for the low VEGF expression group and high VEGF expression group (Mann-Whitney, *p* = 0.258).
(TIF)

S3 Fig Volumetric analysis in anaplastic glioma subtypes. No significant differences in the enhancement/necrosis ratio were identified among the three subtypes (anaplastic astrocytoma, AA; anaplastic oligodendroglioma, AO; anaplastic oligoastrocytoma, AOA) of anaplastic gliomas (Kruskal-Wallis, *p* = 0.586).
(TIF)
