# Supplementary figures and images for: Identifying the Association of Contrast Enhancement with Vascular Endothelia Growth Factor Expression in Anaplastic Gliomas: A Volumetric Magnetic Resonance Imaging Analysis
Source: PLoS One. 2015 Mar 30;10(3):e0121380. doi: 10.1371/journal.pone.0121380 (PMC4379034; doi:10.1371/journal.pone.0121380)

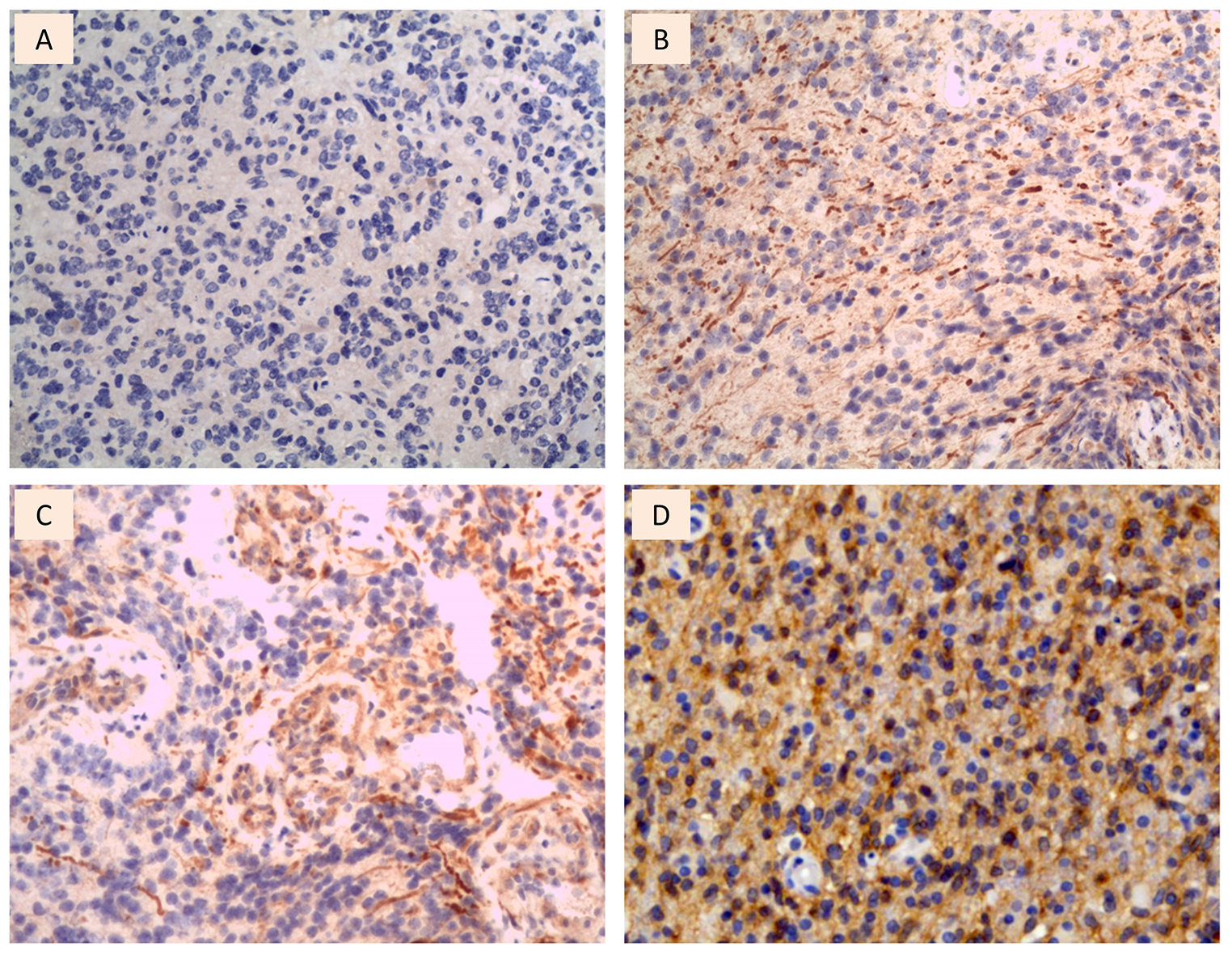

Supplement: S1 Fig — (A) Negative (-); (B) weakly positive (+); (C) positive (++); (D) strongly positive (+++). Magnification: ×200. (TIF) [file pone.0121380.s001.tif]

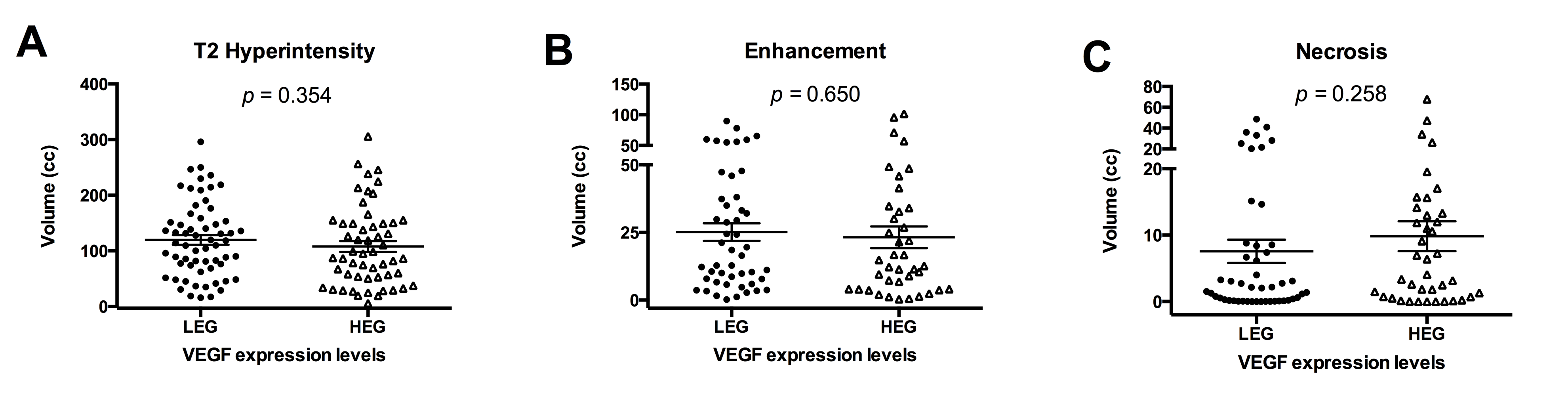

Supplement: S2 Fig — (A) The volumes of T2 hyperintensity for the low VEGF expression group (LEG) and high VEGF expression group (HEG) were not significantly different (Mann-Whitney, p = 0.354). (B) The volumes of enhancement for the low VEGF expression group and high VEGF expression group were not different (Mann-Whitney, p = 0.650). (C) The volumes of necrosis for the low VEGF expression group and high VEGF expression group (Mann-Whitney, p = 0.258). (TIF) [file pone.0121380.s002.tif]

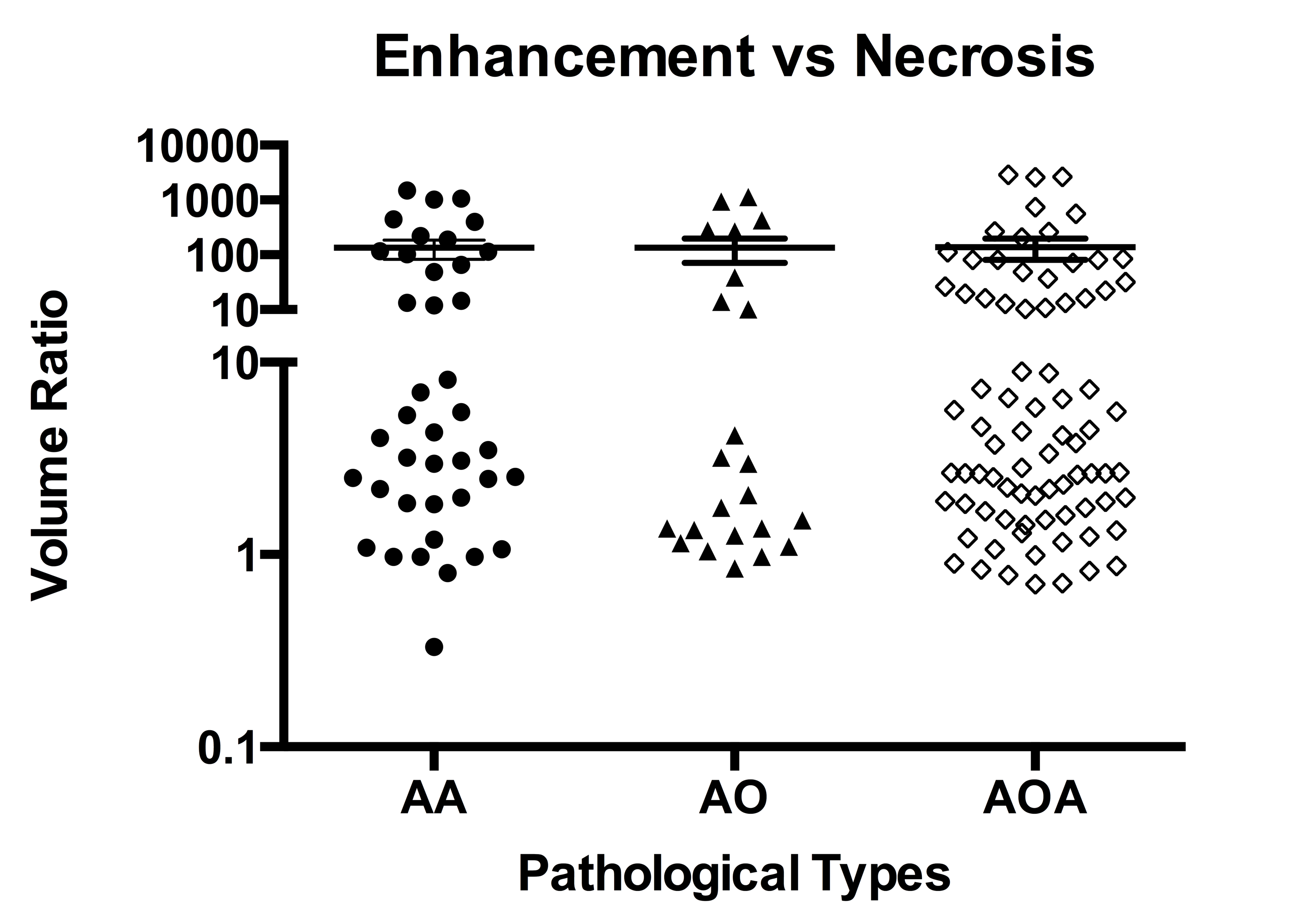

Supplement: S3 Fig — No significant differences in the enhancement/necrosis ratio were identified among the three subtypes (anaplastic astrocytoma, AA; anaplastic oligodendroglioma, AO; anaplastic oligoastrocytoma, AOA) of anaplastic gliomas (Kruskal-Wallis, p = 0.586). (TIF) [file pone.0121380.s003.tif]
